# Preferences for policy measures to regulate urban vehicle access for climate change mitigation

**DOI:** 10.1186/s12302-023-00745-0

**Published:** 2023-06-06

**Authors:** Gabriel Ayobami Ogunkunbi, Ferenc Meszaros

**Affiliations:** Department of Transportation Technology and Economics, Faculty of Transportation Engineering and Vehicle Engineering, Budapest University of Technology and Economics, Budapest, 1111 Hungary

**Keywords:** Car dependency, Policy support, Transport emission, Congestion charging, Emission zone

## Abstract

In cognisance of the urgent need to decarbonise the transport sector to limit its impact on climate change and to internalise other negative transport externalities, regulating vehicle access in urban areas is essential. However, urban areas often struggle to implement these regulations due to concerns relating to social acceptability, heterogeneity of citizen preferences, lack of information on preferred measure attributes, and other factors that can boost the acceptance of urban vehicle access regulations. This study explores the acceptability and willingness to support Urban Vehicle Access Regulations (UVAR) in Budapest, Hungary to reduce transportation emissions and promote sustainable urban mobility. Using a structured questionnaire, which includes a choice-based conjoint exercise, the study finds that 42% of respondents were willing to support a car-free policy measure. Results were analysed to elicit preferences for specific UVAR measure attributes, identify population subgroups, and assess factors influencing willingness to support UVAR implementation. Access fee and proportion of revenue earmarked for transport development were the most important attributes to respondents. The study also identified three distinct subgroups of respondents with differing preferences, which could be characterised based on access to passenger cars, age, and employment status. The findings suggest that for effective UVAR, access fees for non-compliant vehicles should be excluded from measure designs, and the attribute preference approach highlights the importance of considering the heterogeneity of residents’ preferences in UVAR measure planning.

**Supplementary Information:**

The online version contains supplementary material available at 10.1186/s12302-023-00745-0.

## Background

Climate change, driven significantly by greenhouse gas emissions, affects people and ecosystems globally. To mitigate the effects of climate change and avoid the most undesirable consequences, concerted efforts are required across all sectors and levels of society to reduce greenhouse gas emissions. Progress is being made in reducing emission growth rates in many countries and regions in line with the commitment to Paris Agreement and other climate action commitments and pledges. Although significant efforts are still required to achieve the set targets. However, emissions reduction has remained a challenge in sectors like transport [1].

The transport sector is responsible for a quarter of the total final energy used, with 40% of the emissions across all end-use sectors globally, and 90% of this proportion is derived from oil-based sources [2]. In the European Union (EU), transport accounts for about 20% of total greenhouse gas emissions, with more than two-thirds of this accruing to road transport. Within road transport, dependency on passenger cars alone is responsible for 14% of global greenhouse gas emissions. In 2019, road transport accounted for 71.7% of the EU-27 transport sector emissions. Passenger cars dominate road transport modes, accounting for 60.6% of emissions [3]. These trends and the increasing demand for transport and motorisation, particularly in urban areas, make transport one of the most difficult sectors to decarbonise. This is despite the efficiency gains from energy technologies and fuel economy improvements [4]. There have been several calls for demand-side management strategies using a combination of push and pull measures in policy packages [5–7]. Based on its comprehensive assessment report, the Intergovernmental Panel on Climate Change estimated that 20 to 70% of transport emissions could be cut off using more stringent demand-side strategies [8]. However, most policy packages in urban areas targeted at reducing mobility demand and inducing a shift to more sustainable mobility behaviours often lack push measures that could boost these policy packages' overall effectiveness [8]. These policy interventions are considered radical and disruptive and often encounter strong opposition constituting a conflict between political feasibility and environmental policy effectiveness [9]. The transport taboo status of these interventions has consequently created an implementation gap in strategic directions for transport decarbonisation and sustainability [10, 11].

Urban Vehicle Access Regulations (UVAR), such as pricing measures, traffic-regulated zones, and spatial interventions, have been identified as a crucial demand-side strategy for reducing transportation emissions and promoting sustainable mobility in urban areas [12]. Pricing measures can help to reduce energy-intensive transport behaviours by increasing the cost of driving in urban areas and disincentivising private vehicle use. In contrast, traffic regulatory measures and spatial interventions can limit the number of polluting vehicles on the road [13–15]. These regulations can also generate revenues which can be reinvested in sustainable transport infrastructure and services and create more space for improving active mobility [16, 17]. However, it is worth noting that not all the generated revenue may be specifically earmarked for these areas. In some cases, the revenue generated may increase the overall pool of collected tax money [18, 19]. Regulating vehicle access has a plethora of co-benefits beyond climate change mitigation, but it remains a policy approach which is often difficult to implement. As established by many studies, the prospect of air quality improvement, noise reduction, congestion reduction, road safety improvement and an overall improvement in the sustainability and livability of urban areas is often not enough to drive its implementation. Concerns related to social justice, equity, and accessibility, among others, from the public, constitute political barriers [20–23]. They often make these policy instruments missing from the repertoire of many urban areas' sustainable urban mobility measures [24]. When these car usage reduction measures are being considered, determining the policy attributes that citizens and residents will find acceptable and willing to support is another challenge decision-makers must address. Policymakers, therefore, need to consider the trade-offs between environmental policy effectiveness and political feasibility, amongst other factors [25], when designing and implementing these interventions.

To this end, engaging stakeholders and citizens in the decision-making process is essential to ensure the acceptance and implementation of such measures [26]. Public participation could inform the design of such policies and elicit citizens’ preferences for certain policy attributes. Residents' and citizens' acceptance of these measures has consequently been the central theme of many studies evaluating car traffic reduction measures. The studies are often based on identifying factors influencing willingness to accept these measures. [12, 27–30]. However, measure acceptability does not usually translate to a willingness to support.

In the literature on transport policy, acceptability is defined as a construct reflecting individuals' positive or negative attitudes toward a particular policy before its implementation. The notion of acceptance refers to individuals' evaluations of the policy after it has been put into practice. [31, 32]. On the other hand, support goes beyond a potentially passive agreement and includes an active behavioural dimension which spans both before and after the policy's implementation [33]. For instance, support may involve political actions such as calling a representative, gaining signatures on a petition, or voting, while acceptance and acceptability are more passive [34]. While individuals may accept a policy, they may not necessarily support it, as supporting a policy requires a behavioural component that entails a greater opportunity cost [33, 35].

Investigating willingness to support is crucial in policy measure planning [21, 36–38]. In the context of climate change mitigation measures, the importance of examining willingness to support is amplified by the fact that these measures often require significant behavioural changes and individuals willing to advocate for and facilitate their implementation [34, 38]. A study evaluating policy instruments for traffic-related pollution reduction found that individual willingness to take more sustainable modes of transport was a significant predictor of support for the measure [39]. Additionally, an analysis of public support for a carbon tax found that perceived fairness and economic benefits were key drivers of support [40]. Thus, understanding the factors that drive willingness to support, such as attitude to policies, perceived costs or benefits, personal values and behavioural dispositions, is crucial in effectively communicating the benefits of climate change mitigation measures and mobilising public support for their implementation [35, 37, 40].

This study thereby focuses on identifying acceptable policy attributes for urban vehicle access regulations (UVAR) and understanding the factors influencing citizens’ and residents’ willingness to support these measures. It aims to elicit urban residents’ preferences for urban vehicle access regulations by assessing these specific research questions:

- What policy attributes are most acceptable to urban dwellers in relation to UVAR measures?
- How do these acceptable policy attributes vary across different socio-demographic groups?
- What factors predispose urban residents to support urban car-free policy measures?

These questions are addressed by employing a stated preference discrete choice experimental approach using Budapest, Hungary, as a case study. Budapest and indeed Hungary provides an interesting context to investigate the stated questions. The study area has a lower motorisation rate than many other European capital cities and countries. While 47% of the trips in Budapest are made by public transport, 35% by passenger cars and 15% and 2% by walking and cycling, respectively, the transport sector is the second largest source of greenhouse gas emissions in the country after the energy sector [41, 42]. The high emission levels of the transport sector may be attributed to the old passenger car fleet (average age of 15 years) and increasing vehicle miles travelled due to suburbanisation [42]. On the other hand, the recurring appearance of UVAR measures on the policy agenda of Budapest at separate times [43, 44] without an implementation warrants an investigation to identify underlying causes of the transport policy-implementation gap, acceptable policy attributes and pathways to build policy support.

The study combines choice-based conjoint analysis and multinomial logit modelling to identify the important attributes, groups and factors owing to their robustness to account for respondent heterogeneity [45]. The results provide information on the relative importance that urban dwellers ascribe to the policy measure attributes. The combination of the information on the willingness to support the measure and relative weights of attributes and their respective levels can also assist policymakers in designing effective yet acceptable UVAR.

## Method

### Study area

Budapest is Hungary's largest city and capital, with a population of about 1.7 million. Beyond being the political centre, the city is also the country's economic, logistical and cultural hub. With this status, the city has experienced economic growth and infrastructural development. The city also has experienced services development over the decades while offering diverse job, educational and cultural opportunities. These have, however, not been without some attendant challenges similar to the realities of other large urban areas.

On the environmental front, the effects of climate change are becoming increasingly apparent in Budapest with heatwaves due to increasing average temperature and urban heat island effect, flash floods and changing precipitation patterns [46]. Considering the socio-economic impacts of these events on the citizenry, the Municipality of Budapest, in its climate strategy, has undertaken to cut emissions by 40% by 2030 with reference to the 2015 emission level. This target is operationalised into different measures and projects, with the largest cut in CO_2_ emission expected to be achieved by optimising energy usage in buildings and transport [47].

The city aims to increase the share of cycling from 2 to 10%, walking from 11 to 20%, and public transport from 45 to 50% by 2030. At the same time, the share of passenger car traffic is expected to reduce from 40 to 20% [48]. The goal is to reduce traffic and pollution within the urban core by introducing UVAR measures such as climate protection zones that promote public transport, cycling, and electric vehicles. An emission-proportionate congestion charging scheme was included in all planning scenarios of the city's mobility plan, which was adopted in 2019. The congestion charge is planned to be introduced in harmony with other measures, such as low-emission zones and the development of park-and-ride facilities, intermodal centres and central areas of outer districts in an attempt to reduce the necessity of travelling.

The progress towards achieving the set targets, specifically the modal share, experienced a setback in the wake of the COVID-19 pandemic and associated epidemiological measures when public transport ridership dropped drastically. In 2022, the peak workday public transport ridership was only able to reach 83% of its pre-pandemic level in 2019. In contrast, road traffic in 2022 peaked at 110% of the 2019 pre-pandemic workday level [41]. Regardless, the loss in public transport did not imply gains in passenger car mobility only in the city. The conversion of existing car lanes into pop-up bike lanes during this period also influenced an increase in cycling in the metropolitan area [49]. However, the city now has to intensify efforts to minimise passenger car usage and accelerate the city-level reduction of CO_2_ emissions, which has been progressing at an insufficient pace [50].

To address this challenge, the urban transport authority plans to review the mobility plan and re-evaluate the effectiveness of identified measures. Furthermore, the authority aims to realign the strategic policy document with the new sustainable urban mobility planning framework and emerging policy directions for urban sustainability and climate change mitigation in the EU. The strategy review provides a good avenue to re-assess the need to augment the pull measures towards sustainable mobility behaviour with push measures that regulate passenger car usage within the urban area. Coincidentally, a social consultation to re-evaluate the traffic problems and goals in the city was conducted during the same period as the data collection for this study.

### Survey design

A stated preference survey was designed and distributed in the study area to elicit resident preferences in the measure design for urban vehicle access regulations and determine their willingness to support such measures. The survey consisted of four sections with questions having a single choice, Likert-type scale, and open-ended text format.

The first section of the research instrument introduced the study, highlighting its purpose and target respondents. Urban vehicle access regulations were framed in the study as policy instruments to control the access of passenger cars within Budapest and reduce environmental effects and other negative impacts attributed to car dependency in the city. Guarantee of respondent anonymity and responsible data management were provided while respondents' consent to participate in the study was sought with a further option to complete the survey in Hungarian or English.

The next section sought to gather information on the respondents' general travel behaviour, such as their preferred mode for the daily commute and other common trip purposes, and factors influencing their mode choice during trip planning. Respondents were also asked to assess each of the four dominant travel modes (passenger car, public transport, cycling and walking as conveyed in the Budapest Mobility Plan) based on three relevant constructs pre-identified in the pilot phase of the study. Convenience and comfort was selected as a common construct across all modes to provide a possibility for direct comparisons of the modal perceptions. The final question in the section investigated the respondents' perception of general traffic-related problems. This section was designed on the premise that existing travel behaviour and trip planning heuristics like time and cost consciousness largely influence mode choices and potentially policy support. For instance, car owners and drivers are less likely to support access control measures than non-passenger car-dependent respondents [28, 51]. In addition, as identified in previous studies, awareness of mode-specific or general traffic-related problems often determines if the proposed policy instrument will be deemed important and acceptable to the respondents [51–54].

The third section was the stated preference section. To determine how the combination of different measure characteristics influences people's preferences for measures to regulate passenger car access, a choice-based conjoint exercise was developed using Sawtooth Software Lighthouse Studio 9.13.2 [55]. These exercises are widely used in marketing research to examine how variations in product characteristics influence purchase decisions. They have also been adapted to assess preferences in transport decisions [56–58]. The respondents had to complete eight choice tasks showing different policy measure scenarios based on a combination of attributes and levels. The attributes selection to identify citizens’ preferences in designing an UVAR scheme were selected based on criteria characterising existing European schemes (see [59]). Based on good practice of attribute selection, these attributes were chosen because they were identified in a review of UVAR measures; contain elements which affect the social acceptability of the measures; and are instrumental to determining the capital and operation costs (in relation to monitoring and enforcement), hence influencing policy decisions [12, 60]. The attributes include policy coverage area and effective period delineating spatial and temporal limits in which vehicle access will be controlled, vehicles whose access will be regulated in such areas, and the monitoring approach to ensure compliance. Additionally, an attribute to differentiate the measure as a market or non-market-based policy instrument by allowing vehicles into the policy coverage area upon payment of an access fee was included. While another attribute to evaluate the percentage to be earmarked by the urban authorities from such access fees and other policy-related revenue sources to transport development was also added. The attribute levels were selected in such a way as to allow the choice tasks to apply to different cities in Hungary without significant changes and also to reduce the cognitive complexity of the choice tasks for respondents. Four attributes were limited to only two levels, while the others had three. The attributes with their descriptions and levels are presented in Table 1.

**Table 1:** Attributes and levels within the discrete choice experiment

Attributes	Description	Levels
Policy coverage area	If the policy should affect vehicles entering the city centre only or within the significant core of the city (except suburbs and agglomerations)	City centre
		Wider city area
Policy effective period	Days of the week and time of the day in which the policy will be enforced	Monday–Friday (7–19)
		Monday–Friday (0–24)
		Sunday–Saturday (0–24)
Affected vehicles	If the policy should apply to all vehicles (including green plates, i.e. alternative fuelled vehicles) or only vehicles not marked as environment friendly (non-green plates)	Non-green plates only
		All vehicles
Access fee	if drivers of non-compliant vehicles can be allowed to enter the area covered by the policy should be subject to the payment of a fee or not	No
		Yes
Revenue allocation to transport development	What percentage of money generated from access fees, fines, or other payments should be used for transport development	Less than 25%
		25–75%
		Greater than 75%
Monitoring	if compliance monitoring be based on an honour system using stickers or labels with random inspections (manual) or should the systems be monitored automatically using advanced technologies, e.g. camera based systems (automated)	Manual
		Automated

**Fig. 1:**
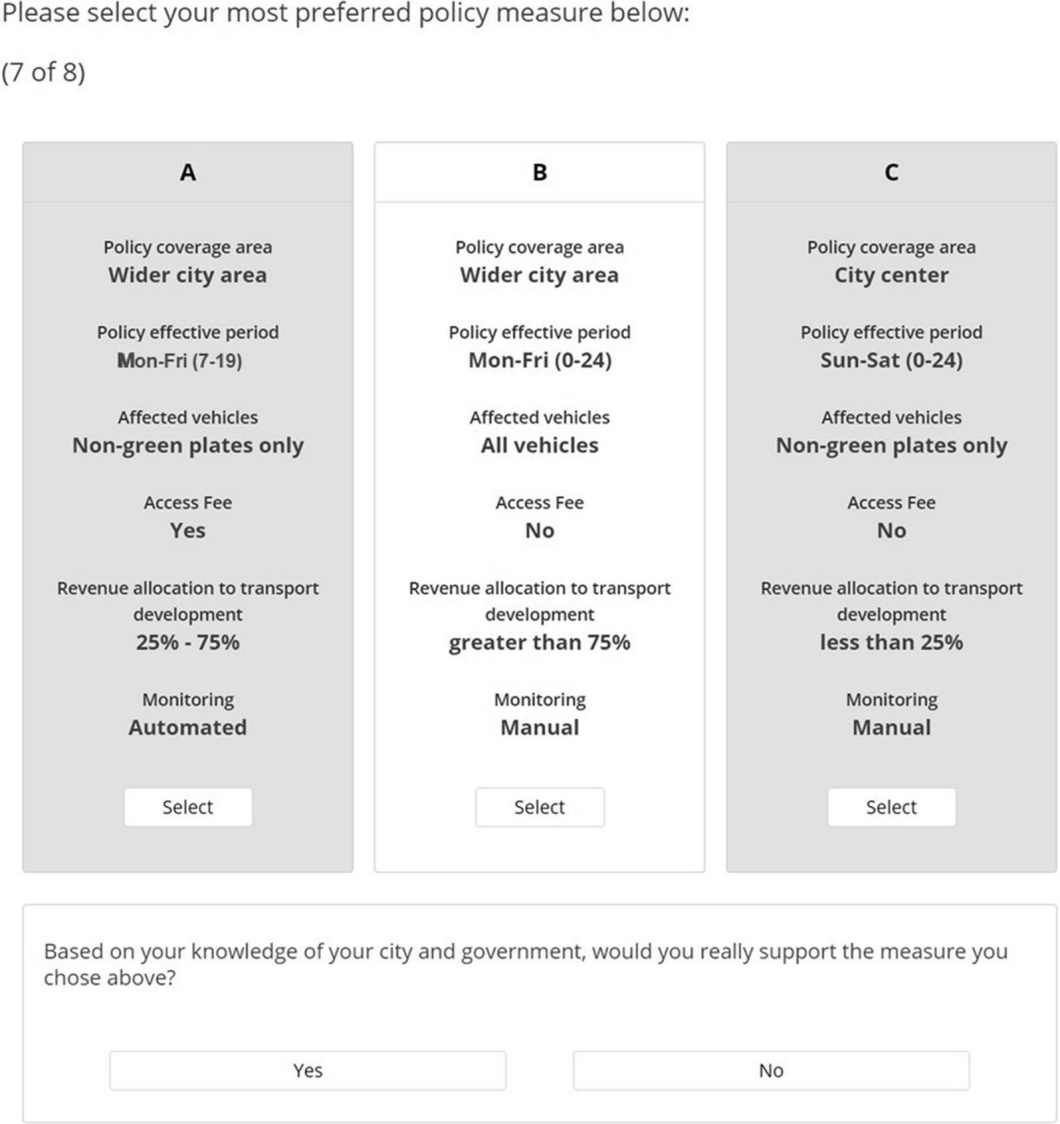
Example of a choice set

The choice task section started with a description of the tasks and the expectation from the respondents. The task descriptions were then followed with an instruction to respondents to choose their preferred measure design out of three policy measure alternatives presented in each task. The opt-out option was not incorporated directly into the alternatives. Rather, it was presented as a follow-up to each of the choice tasks asking respondents if they would support the selected measure based on their knowledge of the mobility conditions and governance of the study area. We adopted this approach to create a compromise between getting more thoughtful responses, which rise from the forced choice method, and reducing bias in parameter estimates [61, 62]. Hence, providing a robust data to compute part-worth utilities from the entire analytic sample while the part-worth utility of the opt-out options (or NONE) were computed separately. Using the Sawtooth software choice-based conjoint balanced overlap feature, we created 300 versions of the experiment with eight choice tasks in each. The balanced overlap design was selected because it guarantees a higher D-efficiency than the shortcut or random designs [63]. Each respondent, therefore, had to make eight choices across 24 presented concepts. A sample choice task is presented in Fig. 1. The section ended with a group of questions where respondents were asked to identify possible behavioural changes or policy reactions if the measures are implemented with items ranging from adopting more sustainable travel modes, modifying trip routine to participating in a protest against such implementation.

The final section of the survey targeted respondents’ socio-demographic characteristics with questions including age, gender, educational level, employment status, area of residence and typical commute destination within the city and income levels. These items were placed at the end of the survey to limit the possibility of anchoring bias and stereotype threats often common in stated preference studies [64, 65]. Beyond the socio-demographic questions, there was a question for respondents to self-identify their willingness to support the adoption and implementation of urban vehicle access regulations in the city. The section and the survey concluded with an open-ended request inviting respondents to provide additional comments regarding their choices or other feedback relevant to the research goals.

### Data collection

The designed survey was hosted on Sawtooth Software servers and was available in English and Hungarian. Two pilot survey rounds were conducted with colleagues and associates within and outside the transport and urban planning sectors to identify characteristics of the dominant travel modes as earlier stated and to pre-test the ease of use, relevance, and comprehensibility of the different parts of the survey. Subsequently, the survey was administered between May and July 2022. A random sampling approach targeting residents of Budapest was employed using online platforms for dissemination. This approach was corroborated with self-selection sampling, as the respondents were invited to decide to participate in the survey before answering the questionnaire and at the time of submission. No personal identifying information was collected, and answers to the open-ended question were anonymised before analysis.

### Analyses

Descriptive analysis was performed on the sample to summarise the distribution across all variables and to identify associations within the characteristics and the stated support for UVAR adoption using IBM SPSS Statistics 29 [66]. Using factor analysis and multinomial logistic regression model functions of the same statistical tool, we identified the likely determinants of the stated support for the policy measure adoption. Preferences for the policy measure attributes and levels were determined by estimating their importance scores and part-worth utilities using the Hierarchical Bayes procedure for the general sample and subgroups (identified with latent class analysis) using Lighthouse Studio 9.13.2.

Factor analysis is used as a dimension reduction technique and latent variables identification [67]. The extracted factors describe the variability within observed and correlated variables in terms of a lower number of unobserved variables and estimate latent constructs of respondents’ attitudes and perceptions. In this study, principal component analysis was selected to reduce the data to an appropriate minimum with the number of factors decided based on eigenvalue > 1. Before the analysis, the data were tested for sampling adequacy and strength of the relationship among variables using the Kaiser–Meyer–Olkin (KMO) test and Bartlett’s test of sphericity, respectively. The results were satisfactory, as KMO values were greater than 0.5, and Bartlett’s test of sphericity was significant (see Tables 4 and 5) [68]. Factors were extracted using Promax rotation, as it produced a simpler structure of loadings and allowed the correlations observed in the factor loadings. Cronbach’s alpha test was subsequently conducted to assess the reliability of the extracted factors.

Due to the discrete nature of the dependent variable of interest, i.e. support for UVAR adoption (yes, no, indifferent), a multinomial logit model was estimated to identify and analyse the factors significantly affecting support for UVAR measures, including the inputs from the factor analysis and other independent variables. The backward stepwise approach was applied to exclude less significant factors and increase the overall estimator efficiency. Insignificant variables with *p* > 0.15 were removed from the model. The removal probability was higher than the more common *p*-value threshold of 0.05 to prevent a loss of important correlates [69].

The utilities and average importances were determined using the hierarchical Bayes method, as it estimates individual-level utilities while preserving the heterogeneity of the population [70]. The method has also been proven to be efficient with choice-based conjoint experiments [71, 72]. Preliminary iterations were run until convergence, and 20,000 draws were made per respondent. The goodness of fit of the conjoint model was assessed using the root likelihood (RLH). As described by [73], an acceptable RLH should be greater than the uninformed probability of choosing an alternative in a choice task. With three policy measure concepts in each choice task of this study, RLH should be greater than 0.33.

We performed a latent class analysis to investigate if distinctive subgroups within the general sample population have varying preferences for UVAR measures. The latent class models were estimated for up to five subgroups, using fifteen replications with different starting values to reduce the risk of suboptimal solutions while retaining the best replication for each model [74, 75]. A three-group solution was selected based on model fit parameters, relative group size and the interpretability of the results [76, 77]. However, to preserve the continuous distribution of heterogeneity in the dataset [75], we estimated the part-worth utilities and average importance of the attributes and levels for each subgroup using the hierarchical Bayes method. We further examined the differences between the subgroups using Chi-square tests.

Recognising the potential effect of our methodical approach and decisions on the outcome of the analysis, we conducted a multiverse analysis. The multiverse of likelihood ratio test estimates for different removal probabilities in the backward stepwise multinomial logit model are presented in Additional file 1: Table A1, while the results of the multiverse analysis for the average importances are presented in Additional file 1: Table A2.

## Results

### Descriptive results

In total, 553 persons accessed the online survey. Eighty-two were incomplete, leaving 471 complete responses. Out of the complete responses, 16 and 44 complete responses were excluded from the analysis due to straight-lining and completion time, respectively, leaving the study with an analytical sample of 409 respondents. To rule on the completion time, we adopted the relative completion speed index approach used by [78], which is obtained by dividing the sample’s median page completion time by the individual completion time. Responses with an index greater than 1.75 were excluded from the study. Table 2 indicates the exclusion of respondents was fairly distributed across the main variable of interest. However, more respondents were removed from those who stated they would support UVAR implementation. The summary statistics of the entire sample are presented in Table 3.

**Table 2:** Distribution of survey responses based on willingness to support UVAR

	Willingness to support UVAR implementation			
	Yes	No	I do not know	Total
Number of complete responses	196	169	106	471
Number of responses in analytic sample	175	141	93	409
Number of excluded responses	27 (13.8%)	21 (12.4%)	14 (13.2%)	62 (13.2%)

**Table 3:** Summary of background characteristics

Characteristic	Frequency (%)	Population data^a^
Age		
18—34	115 (28.1%)	24.3%
35—44	81 (19.8%)	17.3%
45—54	111 (27.1%)	19.0%
55 or older	102 (24.9%)	39.3%
Gender		
Female	221 (54.0%)	53.0%
Male	188 (46.0%)	47.0%
Education		
Secondary education or less	196 (47.9%)	
First degree	115 (28.1%)	
Higher degree	98 (24.0%)	
Paid employment	310 (75.8%)	69.9%
Income		
Less than 200,000 Ft	112 (27.4%)	
200,000–400,000 Ft	215 (52.6%)	
Greater than 400,000 Ft	82 (20.0%)	
Origin		
Near or around the city centre	224 (54.8%)	
Outside the city centre	185 (45.2%)	
Destination		
Near or around the city centre	223 (54.5%)	
Beyond the city centre	186 (45.5%)	
Driving License	280 (68.5%)	
Support		
Yes	175 (42.8%)	
No	141 (34.5%)	
I do not know	93 (22.7%)	

^a^All population data were sourced from Hungarian Central Statistical Office [42]. Numbers represent the Budapest population data except “Age”, which represents national data. The data were enumerated into the presented age groups by the authors. The paid employment data are the employment rate for the population aged 15–74. Income data are provided from by the statistical office in quintiles. However, the average monthly gross income per capita for the year 2021 is estimated at 265, 000 Ft

54% of the respondents are female, and about 52% have attained at least a tertiary degree. About three-quarters of the respondents are fully employed, with more than 70% earning above the gross minimum wage of 200, 000 Ft. More than half of the sample reported living near or around the city centre, and about the same proportion work within the same area. Among the respondents, 175 (42.8%) self-identified as willing to support UVAR adoption and implementation in Budapest, 141 (34.5%) were unwilling, while the others were uncertain about their measure support decision. Comparing the survey sample to the general population data, it can be inferred that the sample is only representative in terms of gender. Additionally, the distribution of the sample is marginally similar to the proportion of the city's residents with paid employment.

**Fig. 2:**
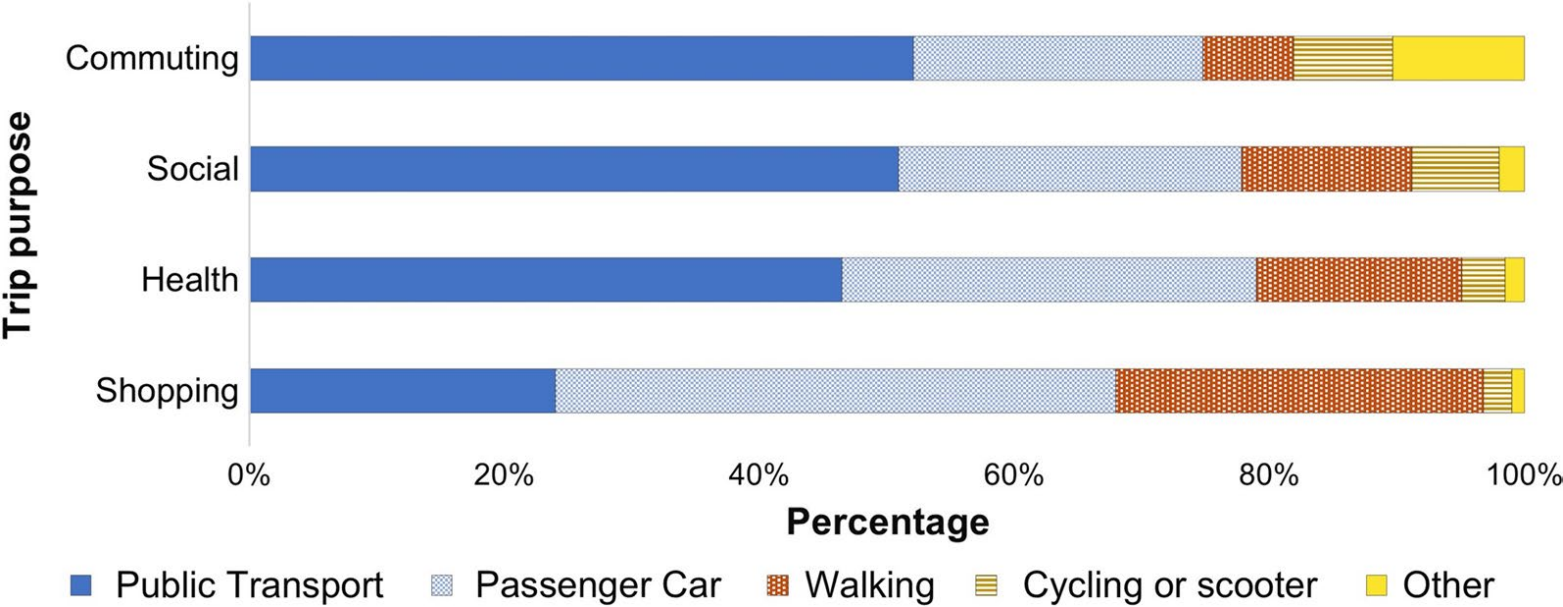
Mode choice across different trip purposes

The frequent modes for urban travel based on different trip purposes, including commuting (e.g. work or school), shopping, health and social trips, were investigated and compared based on the respondent's willingness to support car-free policy measure implementation and adoption. The results are presented in Fig. 2. Despite more than two-thirds of the respondents holding a valid driver's license, public transport enjoys wide usage across all respondents. It is the dominant mode, with a mode share of at least 45% across all trip purposes except for shopping trips, where passenger car travel is the most dominant. A Chi-square test conducted to assess the association between these variables and the willingness to support the UVAR measures established travel mode across all trip purposes to be significantly associated with the choice to support future UVAR measure adoption.

**Fig. 3:**
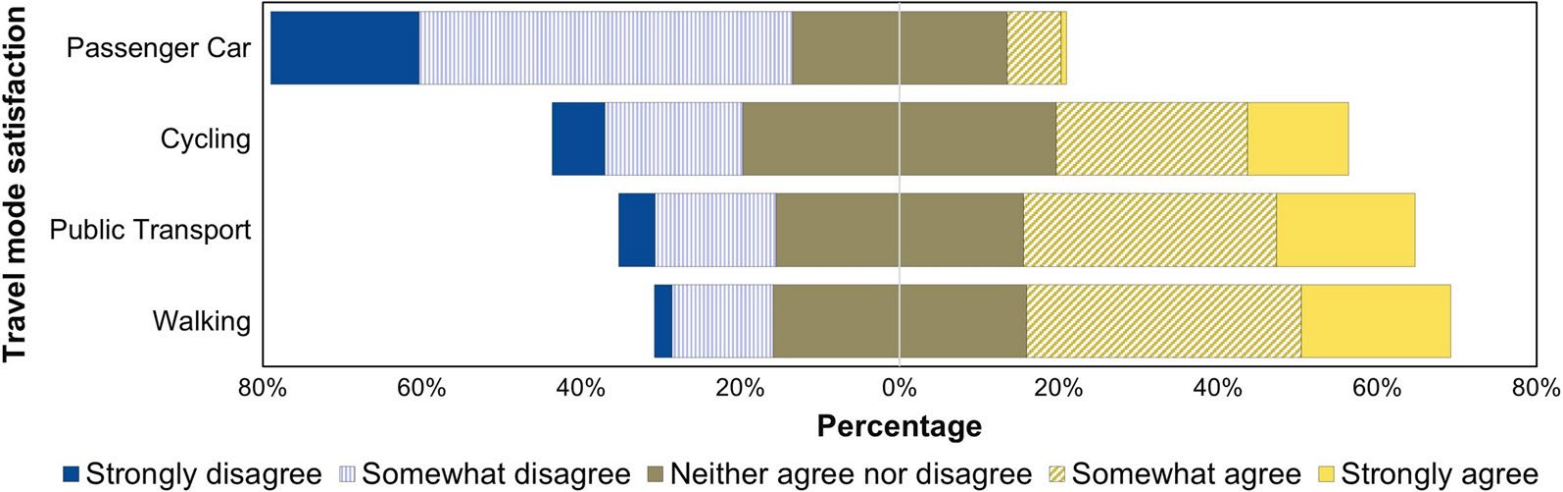
Respondents’ evaluation of travel mode satisfaction

The composite scores of perceived satisfaction with the different transport modes across the valid responses are presented in Fig. 3. It shows that most respondents agreed with statements assessing their satisfaction with walking, public transport and cycling. In contrast, only a minority agreed to be satisfied with passenger car travel within the city. However, the assessment of these scores showed no significant association with the willingness to support UVAR choice.

**Fig. 4:**
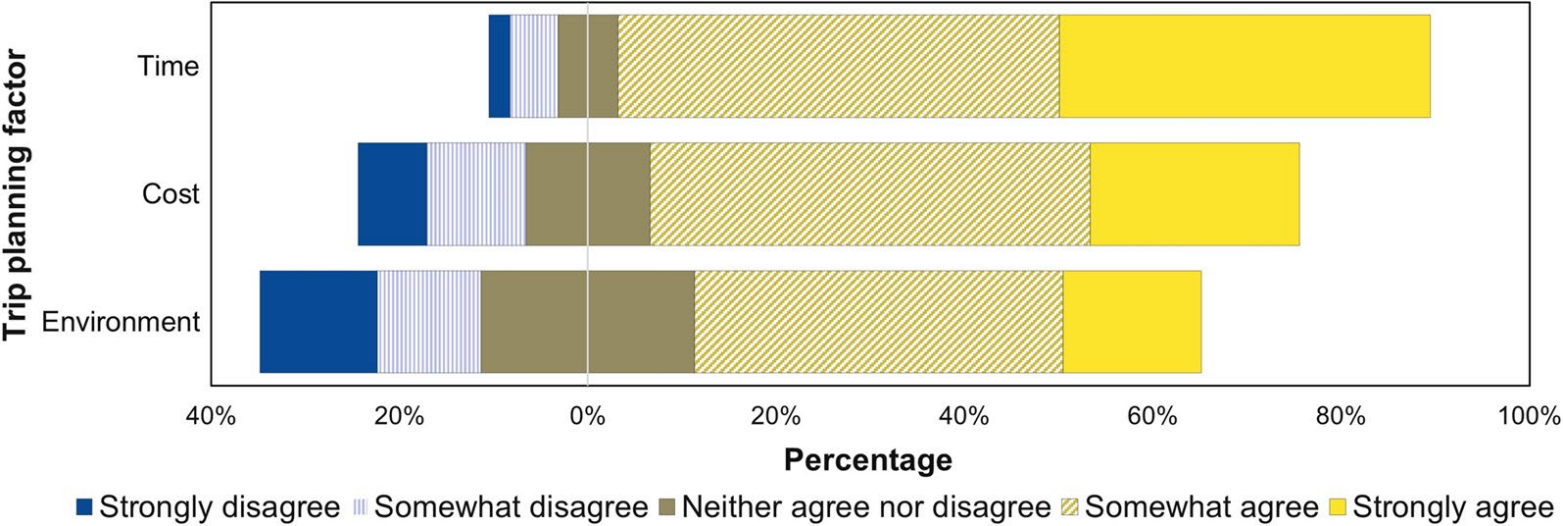
Respondents’ evaluation of factors influencing mode decisions

The final category of items evaluated the factors that influence mode decisions. Figure 4 shows that more than two-thirds of the respondents agreed that cost and time are important to their trip-planning decisions. In contrast, only about half of the respondents agreed that they factored environmental impact considerations into their trip planning. Only the environment variable was found to have a significant association with willingness to support car-free policy measures.

### Multivariate analysis and model development

Exploratory factor analysis was conducted on two different sets of survey items. The first set assessed respondents' concerns with six transport problems in Budapest, with responses collected on a 5-point scale ranging from “not at all concerned” to “extremely concerned”. The outcome of the analysis is presented in Table 4. Three factors were extracted, explaining a cumulative variance of 75% of the total variance with Cronbach’s alpha results indicating a sufficient level of internal consistency [68]. By inspecting the latent meaning these factors might convey, they were interpreted as general transport-related, environment-related, and driving-related problems.

**Table 4:** Factor analysis results for problems perception

Factor	Item	Factor loading	Communality	Cronbach’s alpha
General transport-related problem	Public transport	0.817	0.664	0.72
	Safety	0.790	0.659	
	Active mobility	0.785	0.624	
Environment-related problem	Air pollution	0.957	0.894	0.87
	Noise annoyance	0.908	0.866	
Driving-related problems	Parking	0.940	0.823	0.70
	Congestion	0.782	0.747	
KMO’s measure of sampling adequacy	0.703			
Bartlett’s test of sphericity	Chi-square = 928.637; df = 21; p < 0.001			

The other set of survey items examined the respondent’s willingness to adapt their mobility behaviour should an UVAR be implemented. Respondents rated their agreement to 7 statements on a 5-point scale ranging from “strongly disagree” to “strongly agree”’. After initial analysis, a variable representing respondents' will to protest the implementation was removed to reduce noise in the data because it exhibited significant cross-loading and the data were re-analysed. Consequently, three factors were extracted, as shown in Table 5. Cronbach's alpha test results indicated a sufficient level of internal consistency, with the factors explaining a cumulative variance of 76%. The three factors are interpreted as intentions to reduce car usage, change travel pattern and modify car usage.

**Table 5:** Factor analysis results for behavioural intention

Factor	Item	Factor loading	Communality	Cronbach’s alpha
Reduce car usage	ShiftPuT	0.915	0.813	0.77
	ShiftActive	0.892	0.809	
Change travel pattern	ShiftPlan	0.949	0.813	0.64
	ReduceTrip	0.733	0.702	
Modify car usage	ShiftShared	0.857	0.720	0.60
	ChangeCar	0.836	0.707	
KMO’s measure of sampling adequacy	0.698			
Bartlett’s test of sphericity	Chi-square = 538.218; *df* = 15; *p* < 0.001			

A multinomial logistic model is subsequently applied to investigate the determinants of support for UVAR measures. Based on the response to the willingness to support UVAR question, respondents who indicated “Yes”, “No”, and “I do not know” were categorised into three groups of “support” (*n* = 176), “oppose” (*n* = 141), and “indifferent” (*n* = 92), respectively. The three groups were considered the dependent variables of the model, with the “indifferent” group used as the reference group. The model results are presented in Table 6. Cost-conscious trip planning, pro-environment trip planning, primary commuting mode, problem perception factors, and willingness to adapt behaviour were found to be significantly associated with the tendency to support UVAR measure adoption (*p* < 0.10). For socio-demographic characteristics, age, gender, and income variables showed significant association with the dependent variable. We did not find a significant association between any of the other socio-demographic variables, possession of a valid driver's license or individual perception of travel mode problems with the willingness to support or oppose car-free policy measures for transport decarbonisation and sustainability.

**Table 6:** Multinomial logit model outcomes regarding support for UVAR adoption (ref. = Indifferent)

Explanatory variables	Oppose		Support	
	Coefficient	S.E	Coefficient	S.E
Intercept	0.10	1.13	0.21	1.12
Age (ref. = 55 or older)				
18–34	0.12	0.47	1.19**	0.45
35–44	− 0.43	0.50	0.62	0.46
45–54	− 0.37	0.43	0.45	0.42
Gender (ref. = Male)				
Female	− 0.43	0.34	− 1.06**	0.32
Income (ref. = greater than 400, 000 Ft)				
less than 200, 000 Ft	1.075*	0.53	1.11*	0.51
200, 000–400, 000 Ft	− 0.153	0.42	0.07	0.40
Commuting (ref. = Other)				
Private Car	1.87**	0.65	1.42*	0.62
Public Transport	1.31*	0.56	1.18*	0.52
Walking	1.42	0.82	1.94*	0.81
Bicycle or scooter	1.54	0.83	1.47*	0.74
Planning cost (ref. = Strongly agree)				
Strongly disagree	− 0.01	0.79	0.76	0.71
Disagree	0.33	0.62	0.53	0.57
Neither agree nor disagree	0.22	0.54	− 0.67	0.52
Agree	1.13**	0.43	0.54	0.40
Planning environment (ref. = Strongly agree)				
Strongly disagree	1.35	0.76	− 0.14	0.72
Disagree	− 0.39	0.70	− 1.18	0.62
Neither agree nor disagree	− 0.03	0.64	0.31	0.54
Agree	0.42	0.56	− 0.59	0.47
Protest (ref. = Strongly agree)				
Strongly disagree	− 1.61	0.89	− 0.27	0.92
Disagree	− 2.75**	0.91	− 1.22	0.92
Neither agree nor disagree	− 2.25**	0.85	− 1.73	0.90
Agree	− 0.40	0.93	− 2.11*	1.04
Problem perception				
Environment-related problems	− 0.19	0.20	0.49**	0.19
Driving-related problems	0.43**	0.21	-0.36	0.18
Behavioural intention				
Reduce car usage	0.10	0.20	0.41*	0.19
Change travel pattern	− 0.46**	0.18	− 0.07	0.18
Modify car usage	− 0.35	0.18	0.11	0.18
Summary statistics				
AIC	749.980			
BIC	974.749			
McFadden *R*^*2*^	0.269			

^*^p < 0.05,

^**^p < 0.01,

^***^p < 0.001

The model suggests youths are more likely to support car-free measures compared to older age groups. This is in line with expectations as this age group is often found in studies to be less car-dependent [79, 80]. Low-income earners (earning less than 200,000 Ft) are more likely to support or oppose UVAR measures than higher-income earners. A plausible explanation is that they have lesser financial flexibility to adjust their mobility behaviour in response to changes in transportation costs and access to personal vehicles [81]. Female respondents are more likely to be indifferent towards UVAR measures rather than be willing to support them than male respondents, indicating that transport is gender-sensitive. Regular commuters across all travel modes considered in the study are more likely to have a stance about UVAR policy measure support rather than be indifferent compared to the other category (including those who do not commute often). Nevertheless, the data did not find a significant relationship to suggest that people who use active travel modes will oppose adopting such measures. However, people who agree to be cost-conscious trip planners are more likely to oppose the measures.

Based on the perception of general transport problems in the city, people more concerned with driving-related problems like congestion and parking are more inclined to oppose car-free policy measures. It could be assumed that people in this category are more concerned about these problems due to their car-oriented lifestyles and will be unwilling to give up their passenger car usage. In contrast, people conscious of environment-related problems will be willing to see the city become less car-dependent to reduce noise and emissions.

Regarding willingness to adapt behaviour, the results suggest people who declared intention to switch to sustainable travel modes or change their travel patterns are more likely to support the policy measures. As expected, people who disagree or stay neutral about protesting the measures are less likely to oppose the measures.

### Preferences for UVAR measure design

**Fig. 5:**
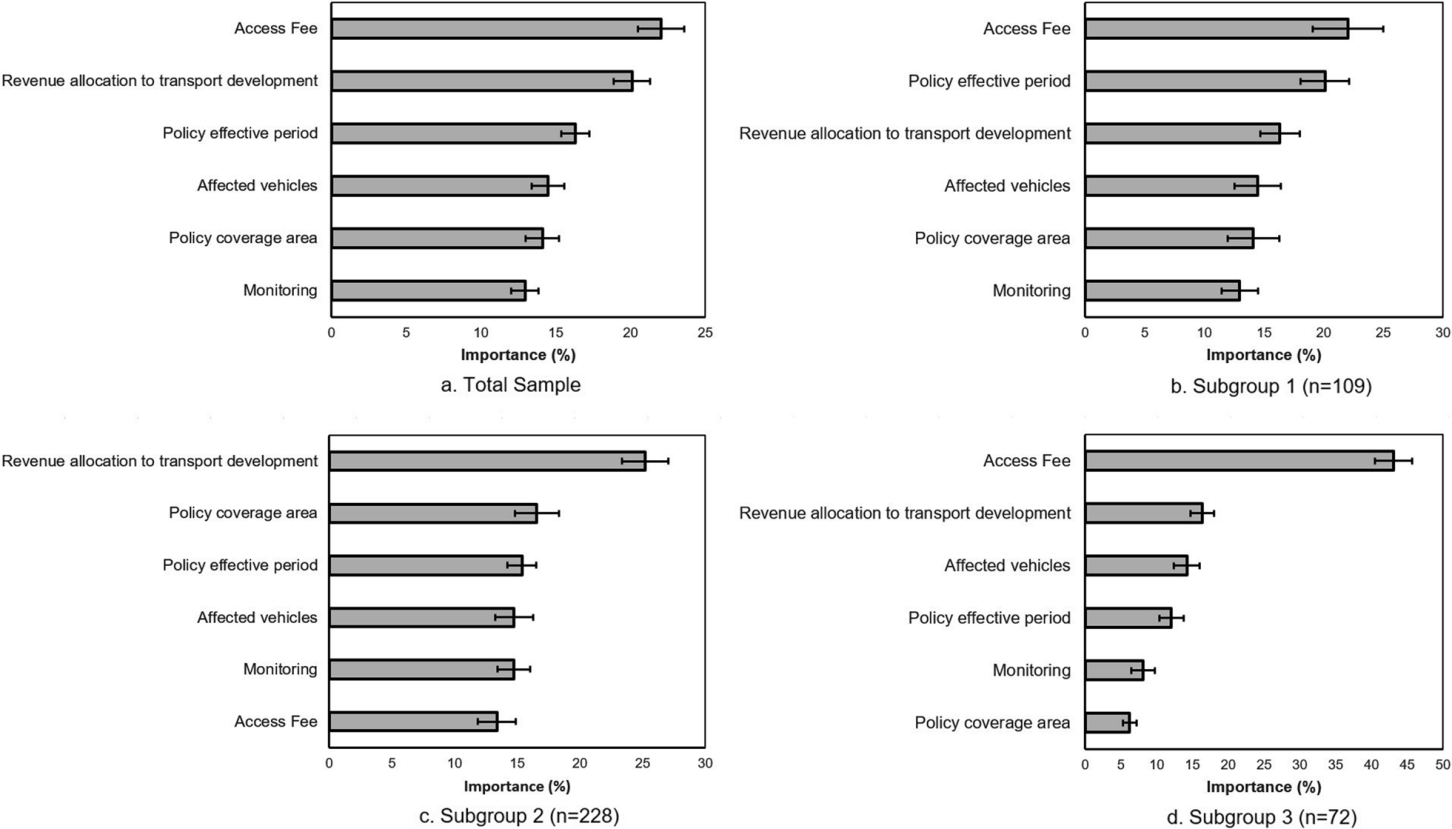
Importance of the measure attributes in the total sample and subgroups

Based on the analysed responses from the total sample, the availability of an access fee option to the regulated area was the most important UVAR attribute (22.14%), followed by revenue allocation to transport development (20.75%) (see Fig. 5a). However, there was no statistical difference between these two. These were followed by policy effective period, affected vehicles and policy coverage area, with the average importance of the latter two not being statistically different. Monitoring was evaluated as the least important attribute scoring an average importance score of 12.72%.

The part-worth utilities and the respective confidence intervals (CI) are presented in Table 7. The result shows that the participants preferred vehicle access to be regulated within the city centre for all vehicle types with no option for an access fee for non-resident vehicles to enter the regulated area. Working hours on weekdays were the preferred policy effective period with an automated monitoring method. For revenue allocation to transport development, there is no significant difference observed between earmarking 25–75% of the revenue generated for transport development and greater than 75%.

**Table 7:** Part-worth utilities of the measure attribute levels

	Part-worth utility	Lower CI	Upper CI
Policy coverage area			
City centre	18.05	13.09	23.02
Wider city area	− 18.05	− 23.02	− 13.09
Policy effective period			
Mon–Fri (7–19)	18.49	13.26	23.72
Mon–Fri (0–24)	− 4.68	− 7.69	− 1.68
Sun–Sat (0–24)	− 13.81	− 18.72	− 8.89
Affected vehicles			
Non-green plates only	− 26.99	− 31.55	− 22.43
All vehicles	26.99	22.43	31.55
Access fee			
No	21.97	14.31	29.63
Yes	− 21.97	− 29.63	− 14.31
Revenue allocation to transport development			
Less than 25%	− 49.81	− 55.49	− 44.13
25–75%	22.29	19.74	24.84
Greater than 75%	27.52	21.79	33.25
Monitoring			
Manual	− 31.36	− 34.83	− 27.89
Automated	31.36	27.89	34.83
None	− 162.45	− 204.55	− 120.34
Fit statistic (RLH)	0.61		

Part-worth utilities should be compared within an attribute

### Identification of latent subgroups

The latent class analysis revealed three subgroups with different preferences for UVAR measure design. The importance of the investigated measure attributes is shown in Fig. 5b–d, and the part-worth utilities are presented in Table 8. The differences in the socio-demographic and dependent variables within the groups are presented in Table 9. For subgroup 1 (*n* = 109), access fee (22.34%) and policy effective period (21.37%) were the most important attributes, for which the importance score did not differ significantly from each other. These were followed by revenue allocation to transport development (15.21%), affected vehicles (13.97%) and policy coverage area (13.71%). The least important attribute of the group is monitoring, with an average importance of 13.40%. Similar to the findings within the total sample, a policy effective period of Weekdays (7–19) with no possibility of an access fee was favoured. The subgroup will prefer a policy affecting all vehicle types within the city centre, which is monitored automatically for compliance, with about 25–75% of all revenue earmarked for transport development. Members of this subgroup have a greater tendency to be unwilling to support a car-free measure in urban areas, which is further confirmed by the high utility value observed for their "None response" in the dual response choice tasks.

**Table 8:** Part-worth utilities of the measure attribute levels across the subgroups

	Part-worth utility (95% confidence interval)		
	Subgroup 1	Subgroup 2	Subgroup 3
Policy coverage area			
City centre	16.25 (6.64, 25.85)	27.33 (19.79, 34.87)	− 12.87 (− 17.17, − 8.57)
Wider city area	− 16.25 (− 25.85, -6.64)	− 27.33 (− 34.87, − 19.79)	12.87 (8.57, 17.17)
Policy effective period			
Mon–Fri (7–19)	46.60 (35.06, 58.15)	7.55 (0.56, 14.54)	5.05 (− 2.59, 12.68)
Mon–Fri (0–24)	− 4.94 (− 12.52, 2.64)	− 8.17 (− 12.18, − 4.17)	5.70 (− 0.72, 12.13)
Sun–Sat (0–24)	− 41.67 (− 50.16, − 33.17)	0.62 (− 5.56, 6.81)	− 10.75 (− 20.93, − 0.57)
Affected vehicles			
Non-green plates only	− 19.68 (− 28.79, − 10.57)	− 24.30 (− 30.96, − 17.63)	− 42.29 (− 47.82, − 36.75)
All vehicles	19.68 (10.57, 28.79)	24.30 (17.63, 30.96)	42.29 (36.75, 47.82)
Access fee			
No	49.27 (36.92, 61.63)	− 31.32 (− 36.88, − 25.76)	129.28 (121.56, 136.99)
Yes	− 49.27 (− 61.63, − 36.92)	31.32 (25.76, 36.88)	− 129.28 (− 136.99, − 121.56)
Revenue allocation to transport development			
Less than 25%	− 26.42 (− 35.50, − 17.33)	− 71.15 (− 78.83, -63.47)	− 12.26 (− 24.40, − 0.11)
25–75%	20.57 (14.58, 26.55)	25.27 (20.83, 29.72)	11.87 (7.73, 16.02)
Greater than 75%	5.85 (− 1.93, 13.63)	45.87 (37.91, 53.84)	0.39 (− 12.19, 12.97)
Monitoring			
Manual	− 16.33 (− 24.63, − 8.03)	− 42.82 (− 46.94, − 38.70)	− 12.86 (− 19.73, − 5.99)
Automated	16.33 (8.03, 24.63)	42.82 (38.70, 46.94)	12.86 (5.99, 19.73)
None	387.83 (357.21, 418.45)	− 209.60 (− 226.97, − 192.24)	− 111.32 (− 122.36, − 100.29)
Fit statistic (RLH)	0.65	0.55	0.70

Part-worth utilities are to be compared within one attribute and one subgroup (not across attributes and subgroups)

**Table 9:** Differences across socio-demographic variables between the subgroups

Variable	Group 1 (*n* = 109)	Group 2 (*n* = 228)	Group 3 (*n* = 72)	Chi-square
Commuting				24.327**
Private car	40 (36.7%)	35 (15.4%)	18 (25.0%)	
Public transport	51 (46.8%)	127 (55.7%)	35 (48.6%)	
Walking	3 (2.8%)	19 (8.3%)	7 (9.7%)	
Bicycle or scooter	9 (9.3%)	19 (9.3%)	4 (5.6%)	
Other	6 (5.5%)	28 (12.3%)	8 (11.1%)	
Age				20.624**
18—34	26 (23.9%)	79 (34.6%)	10 (13.9%)	
35—44	27 (24.8%)	44 (19.3%)	10 (13.9%)	
45—54	33 (30.3%)	52 (22.7%)	26 (36.1%)	
55 or older	23 (21.1%)	53 (23.2%)	26 (36.1%)	
Gender				0.435
Female	56 (51.4%)	125 (54.8%)	40 (55.6%)	
Male	53 (48.6%)	103 (45.2%)	32 (44.4%)	
Education				6.463
Secondary or less	47 (43.1%)	110 (48.2%)	39 (54.2%)	
First degree	29 (26.6%)	63 (27.6%)	115 (31.9%)	
Higher degree	33 (30.3%)	55 (24.1%)	10 (13.9%)	
Paid employment	94 (86.2%)	167 (73.2%)	49 (68.1%)	9.638**
Income				5.261
Less than 200,000 Ft	22 (20.2%)	71 (31.1%)	19 (26.4%)	
200,000–400,000 Ft	61 (56.0%)	117 (51.3%)	37 (51.4%)	
Greater than 400,000 Ft	26 (23.9%)	40 (17.5%)	16 (22.2%)	
Origin				1.422
Near or around the city centre	58 (53.2%)	122 (53.5%)	44 (61.1%)	
Beyond the city centre	51 (46.8%)	106 (46.5%)	28 (38.9%)	
Destination				0.019
Near or around the city centre	59 (54.1%)	125 (54.8%)	39 (54.2%)	
Beyond the city centre	50 (45.9%)	103 (45.2%)	33 (45.8%)	
Driving License	92 (84.4%)	137 (60.1%)	51 (70.8%)	20.422***
Support				53.730***
Yes	22 (20.2%)	125 (54.8%)	28 (38.9%)	
No	66 (60.6%)	53 (23.2%)	22 (30.6%)	
Indifferent	21 (19.3%)	50 (21.9%)	22 (30.6%)	

^*^p < 0.05,

^**^p < 0.01,

^***^p < 0.001

On the other hand, subgroup 2 (*n* = 228) had revenue allocation to transport development (25.34%) as the most important attribute, followed by policy coverage area (16.55%) and policy effective period (15.37%), for which there was no statistical difference. The least important attributes were affected vehicles (14.75%), monitoring (14.71%) and access fee (13.38%). Consistent with the general findings, the subgroup preferred that greater than 75% of the revenue generated should be allocated for transport development with vehicle access regulated within the city centre during weekday working hours. The part-worth utilities also reflect that the measure should affect all vehicle types with an automated monitoring system. However, they consider there should be an option for an access fee for non-compliant vehicles. The members of this subgroup exhibited the highest willingness to support a car-free policy within the urban area.

For subgroup 3 (*n* = 72), access fee was the most important attribute (43.09%), with a preference that polluting vehicles are not granted access into the policy area. This was followed by revenue allocation to transport development (16.38%) and affected vehicles. The subgroup will prefer that the policy affects all vehicle categories and will want at least 25% of the revenue to be used to further improve the transport infrastructure and mobility offers within the city. The subgroup favours a weekday scheme with automated monitoring across a wider city area.

### *Qualitativeresults*

A qualitative analysis of the comments made in the optional feedback field of the survey is presented in this section. Fifty-nine comments were received expressing concerns, considerations, and conditions under which implementing an UVAR measure might be practical in the city. The main findings from these comments are summarised in the following:

- **Governance, trust and transparency**

The role of governance and transparency regarding the measure was important to a good number of the respondents. While it can be inferred from the statistical data, a couple of respondents in their comments strongly opined that reducing the city's negative transport externalities is essential. However, they were sceptical about the intentions and capacities of the urban authorities. The sceptical residents want purpose-driven and tailor-made solutions which are not driven by other motivations. Along the same perspective, there were views that the government might introduce such regulations specifically for revenue generation, stating they will only find them acceptable, provided the revenues generated are earmarked for transport development.

*“Find a professional solution, not a politically motivated one, especially in Budapest.”*

*“As we know, the rule of the people is not about the balance of society but about the economic hysteria currently driven by money. If introduced, 100% of the revenue would have to be used for transport development and nothing else.”*

On another perspective, calls for more efforts at integrated planning and improving local accessibility was suggested to be a prerequisite to regulating vehicular access, especially in areas outside the city centre.

*“A much clearer planning of road transport and urban development is needed to first reduce number of trips made before regulating cars.”*

Furthermore, there were views that the government should plan such measures adequately and remain committed to them when implemented.

*“Let there be more planning and let the plans be implemented for [the purpose] which they were made.”*

*“Don’t stop the regulations once you’ve started.”*

b. **Social justice and equity**

Urban vehicle access regulations can raise several social justice and equity concerns amongst residents, and this was a recurring theme in most of the respondents’ comments. The primary issue raised was about the marginalisation effect that the regulations might have on the economically disadvantaged and the elderly in society. Many of these were in light of alternative modes’ substituting capacity to cater to the needs of those who can no longer use their cars. Many of the viewpoints were supportive and expressed an understanding of the need for the measures.

*“It is important to protect our environment, but as long as most of the population is currently spending so much money to meet domestic needs, no one will be able to afford and switch to zero emission cars.”*

*“If you want to restrict access to the downtown, you should NOT be able to get out of the rules by paying. It gives an unjustified advantage to the ‘money’ road users.”*

Their concerns were, however, founded as a respondent confirmed their fears.

*“Let there be a toll for everyone. I will pay it, I will not change my car or the use of my car because of it.”*

On the other end, opposing views about such regulations were also made, with a particular viewpoint stressing that taking space for cars away is equally inequitable.

*“I can only support something that is free of extremes and takes into account of all people, not a particular group.”*

*“Cycling and walking also depend on age and physical fitness. While it is good to encourage it, it can only be done at a younger age … it would do more harm than good. This should be equally considered.”*

*"It is nonsense to make Budapest car-free. The city was not built in the old days to convert car lanes for bicycles. Not everyone is young enough to get on a bicycle or scooter. We need flyover systems or separate facilities to direct cycling traffic."*

iii. **Transport infrastructure and service improvement**

There appears to be a consensus among respondents that improved transportation options are necessary for vehicle access regulations to be effective. Specifically, some emphasised the importance of investing in transport infrastructure to accommodate or reroute passenger car inflow that cannot access the city and improve active mobility.

*“It’s also important to create the necessary infrastructure to take away the displaced traffic.”*

*“It is not yet time to go car-free—neither the capital, roads, nor the transport facilities are suitable—it would only add to the chaos … and bicycles are downright dangerous on these potholed roads and in heavy traffic.”*

Other opinions were directed towards making public transport more attractive and efficient in the city.

*“My view is that, in terms of transport, we should definitely prioritise public transport and make it of a quality and value that makes it worth choosing. For short distances, walking may be the preferred option. I'm not sure about cycling since I don't use it but it should be in the mix also.”*

*“Why force those who cannot replace their cars for financial reasons to use the inefficient public transport system instead of their good old cars?”*

*“… until there is adequate public transport, these measures should not be introduced.”*

d. **Incentives and complementary measures**

The need for incentives and other complementary measures to minimise the effect of regulating passenger car usage is another theme where the opinions of most respondents converge. These opinions were expressed particularly concerning switching to zero-emission vehicles.

*“At present, only the rich use electric cars, which are not known to be totally environmentally friendly. The lower classes are not buying new modern cars because they do not want to, but because they cannot afford them. This is an area where the government should support more.”*

*“Banning cars from the city centre is a logical step if the average person can convert their car to electric. At the moment, this is not a given, nor is the extra cost of transport in such a case. If these needs are met, there is a realistic chance for the measures.”*

Other areas with fewer mentions will be the further development of park-and-ride options for passenger car inflow from agglomeration areas of the city and improved pricing and service models from shared mobility providers.

*“I live outside the city and I rarely go downtown. Even then, I will always stop and park my cars at parking garages, as long as I can easily make a transfer to public transport.”*

*“Passenger cars will be needed for some trips and much cheaper green car-sharing schemes should be developed for that.”’*

## Discussion

In cognisance of the urgent need to decarbonise the transport sector to limit its impact on climate change and to internalise other negative transport externalities, regulating vehicle access in urban areas is essential. However, urban areas often struggle to implement these regulations due to concerns relating to social acceptability, heterogeneity of citizen preferences, lack of knowledge of measure attributes, and other factors that can boost the acceptance of urban vehicle access regulations. This present study adopts a stated preference discrete choice experiment to uncover this information for Budapest, with broad insights for other urban areas. The results indicate that a simple majority will be willing to support the implementation of a car-free policy measure in the city. Nevertheless, the high proportion of indifferent or sceptical respondents (about 23%) implies the outcome could sway in the opposite direction if measures are designed without adequately considering the preferences and needs of all stakeholders.

As expected, higher utility or part-worth values are ascribed to the less-restrictive attribute levels to passenger car access as expressed in the preferences for a measure covering the city centre and active during weekday working hours only. This indicates the quest to retain the perceived freedom enjoyed in the absence of active access regulation of passenger cars within the city. However, the hierarchy of importance ascribed to the attributes is an interesting finding. Of notable mention is the importance ascribed to access fees in the general respondent groups and the latent subgroups. It confirms the sensitivity of the populace to the generalised cost of travel. Although, a preference for an access fees option in line with self-interest maximisation was expected in the study. The anticipated compromise allows for flexibility, as citizens who cannot switch to sustainable travel modes or change to zero-emission vehicles still have vehicular access upon the payment of a stipulated fee.

The second most important attribute offers a solution to this stalemate. With a large proportion of the revenue generated from the measure implementation dedicated to financing transport development, the city administration can significantly improve transport offers and defragment public transport connections in the city. could be significantly improved. This will be particularly vital for residents living in the peripheral regions of the urban area who will need enhanced sustainable travel options in the instance of regulated passenger car access. At the same time, exemptions can be introduced within a limited period to cater to the special mobility needs of those who depend on passenger cars for daily commuting.

However, the preference of rejecting access fee as a second-best policy option brings to the forefront that the respondents mostly identify such an option will create a loophole that may lower the effectiveness and efficacy of the proposed policy measure. The finding shows that an access fee for car-free policy measures will mostly impact people in lower income groups, who may be unable to afford the fee. But, it might not discourage people in higher income groups from continuing unsustainable mobility behaviour.

Similarly, the results highlight an overall preference for a policy measure that affects all vehicle classes equally. This is without excluding green cars despite their lower emission impacts—a highly radical measure if applied to a wide urban area. The preference can be inferred to be a case that a measure affecting a population segment may be considered discriminatory or unfair and can widen the gap between high and low-mobility population segments [82]. Moreover, with the respondents' arguments, electric vehicles are currently affordable to high-income households who often prioritise time over cost. Preferential vehicle access may imply increased speeds, which can negatively affect the safety of other road users and urban air quality (from non-exhaust particulate matter) if unchecked. Other possible effects are accelerated battery degradation and increased energy consumption, which can ultimately increase well-to-wheel emissions [83].

The other goals of the study are to investigate if distinct segments of the sample with differing preferences for car-fee policy measure design exist and whether these segments can be characterised based on their travel behaviour, perceptions and socio-demographic characteristics. Three subgroups were identified from the analysis. Two subgroups (1 and 3) based their choices on the absence of access-fee in the policy measure, while the third group prioritised the proportion of revenue dedicated to transport development.

In addition to the access fee attribute, greater preference was also stated for the policy effective period in Subgroup 1. Subgroup 1 has the largest share of respondents with valid driver's licences and those who depend on passenger cars for their daily commute but has the lowest proportion of unemployed samples. The subgroup also has the lowest part-worth utility in the dual response option of the choice tasks, which is consistent with the finding that the larger proportion of the respondents in the subgroup declaring not to support the implementation of a passenger-car reduction measure in the city. This implies that the subgroup constitutes most respondents who doubt the government's capacity or commitment to implement the policy measures effectively. As this can be inferred to be a lack of trust in governance capabilities, it extends previous findings, which identified trust as a vital construct to the acceptability of urban vehicle access regulations and stringent policy measures [84, 85]. The resistance could also be explained by the dependence of the more significant share of the subgroup on passenger cars for their mobility needs. This, therefore, positions the subgroup as critical to the success of transport decarbonisation efforts. A policy measure based on the attributes and levels that maximise the subgroup's part-worth utilities may suggest a policy measure alternative likely to face the least resistance. It could therefore be the basis for introducing an UVAR measure. This measure can subsequently be improved or expanded after implementation based on the empirical evidence that acceptability and support improve post-implementation of UVAR measures [27, 28, 86].

Subgroup 2, consisting of about 55% of the respondents, did not prioritise access as an attribute of importance in their choice decisions, unlike the other subgroups. Additionally, the subgroup favoured the introduction of an access fee for non-compliant vehicles. The subgroup members based their choices on earmarking a higher share of the revenue generated for transport development. Unsurprisingly, they favoured that an access fee is imposed on drivers of non-compliant vehicles to enter the policy coverage area. A possible explanation is that 85% of the subgroup do not use passenger cars for their daily commute, with 56% depending on public transport for this trip purpose. Interestingly, more than half of the subgroup population is willing to support the implementation of UVAR. This supports the findings that young people and public transport users are more predisposed to support policies that promote lifestyles that are less dependent on passenger cars, provided public transport and other complementary measures are developed for optimal service delivery and use.

Subgroup 3 has the closest similarity to the preferences of the general sample, except that the most important and least important attributes significantly differed from others. This subgroup which uniquely favoured an UVAR implementation in the wider city area comprised the highest proportion of respondents older than 44 years old and without a paid job (as a good number were retirees). Being the most senior group with low daily commuting needs, they probably will not be affected by passenger car regulations. They can afford to be flexible with the schedule of their trips. Notwithstanding the indifference exhibited by the subgroup, their defining characteristics make them important. With about 70% of the subgroup having a valid driver's license, they have the tendency to be social car users and are capable of fulfilling their mobility needs through other modes. They will therefore be a good target population for awareness campaigns. At the same time, older people's tendency for pro-sociality as suggested by [87], which positions them with the likelihood of being able to influence social norms and values towards sustainable mobility behaviour, could be explored.

The multinomial logistic model results further validate the findings of the choice-based conjoint analysis. It identifies people of the lowest age and income group, dependent on public transport or active mobility for their daily commute, as more likely to support the implementation of UVAR measures. It also suggests that residents more concerned with the environmental effects of transport in the urban area are likely to support the measures. Expectedly, urban dwellers willing to reduce their car usage or change their travel patterns are also identified to be pro-UVAR. However, the interesting finding from the model is that people concerned with congestion and parking are more likely to oppose the measures. More so, female residents are less likely to support car-free policy measures. The former could be explained by positing that their concerns with driving-related problems are insufficient to deter them from their supposed habitual car dependency. There are probably other underlying factors to explain their choice, which goes beyond the individual behaviour and characteristics investigated in this study.

Gender as a predicting factor for mode choice, car-free lifestyle and sustainable transport policy decisions has remained a grey area, with studies arriving at different conclusions. Moreover, safety and security concerns coupled with trip-chaining tendencies have been identified previously as factors which may predispose female citizens to favour passenger car travel [88, 89]. Therefore, the finding re-emphasises the importance of factoring gender-based considerations into urban vehicle access regulation planning and other transport policy decisions [90, 91].

The qualitative analysis puts the entire study into a broader perspective. It identifies path dependencies and lock-ins associated with passenger car usage [92]. This needs to be overcome if the goal of decarbonising the transport sector and making urban areas more livable will be reached [93]. Building support for urban vehicle access regulations to achieve the maximum climate change mitigation potential will require concerted efforts from all stakeholders to overcome the behavioural, institutional, and infrastructural lock-ins identified in this study. This will include raising awareness about co-benefits associated with active travel, sensitisation efforts geared at decoupling private car ownership from social identity and promoting shared mobility. It will also require transport service integration to encourage intermodality, sustained policy support for environment-friendly mobility and cross-sectoral urban development. Additionally, the specific concerns on the non-affordability of alternative fuelled vehicles and the potential disproportionate impact of UVAR measures, particularly on low-income earners, further stress the need for Hungarian policymakers to ensure the transition to more sustainable transportation options is accessible to all members of the society. As the concerns might be an indicator, for example, that previous purchase incentives for environmentally friendly vehicles were only effective in promoting the uptake among some segments of the population. However, the current research instrument did not provide an avenue to investigate this further.

By aggregating the discussed attribute levels of the study’s sample, it can be deduced that the urban residents will be more willing to support a low or zero-emission zone than congestion charging which is more prominent in Budapest's planned measures. This, however, should not be interpreted as placing a policy instrument above another, as the study fully acknowledges the efficacy of pricing and other push measures for mobility demand management. Policy instruments often result from place-based decarbonisation strategies built on societal readiness. This outcome is based on the sample's willingness to support and preferences. In addition, implementing an emission zone in Budapest to lower transport carbon emissions and combat climate change is a beneficial approach. This is particularly important as the legality of a congestion charge remains a policy debate in the city and could have informed the view of the research participants.

Like all stated preference surveys, hypothetical bias could have affected the responses, irrespective of our efforts to control it. The study design, notably the choice experiment, required some assumptions and simplifications partly due to the abundance of attributes factored into urban vehicle access regulations measure planning. For example, this study did not include a cost attribute in the choice experiment, which could have allowed the estimation of willingness to pay parameters, but rather simplified with a binary choice of presence or absence of an access fee. We also did not consider other variations in exemptions beyond vehicles affected as the feedback during the pilot phase were divergent. The need to reduce the complexity of the study and minimise survey drop-out rate and completion time prompted us to limit the investigated variables to individual background characteristics and mobility behaviour variables. The attributes and variables that were not included may also be very important and may affect the results to a certain extent. The study could therefore be extended using established sociological and psychological theoretical frameworks to identify such factors. In addition, investigating the preferences of the urban population in cities where passenger car is the dominant mode would be beneficial to the generalisability of the findings of this study.

## Conclusion

The primary outcomes of this study are twofold. First, it identifies access fee as the most impactful attribute in eliciting people’s preferences for urban vehicle access regulations measure design. Many respondents considered granting access to non-compliant vehicles may lower the emission reduction potential of the measures and favour a specific social class. Revenue allocation to transport development comes close, mainly because it aids the further development of passenger car alternatives which is vital in addressing social justice concerns. Secondly, the primary mode for commuting, age, employment status, and ability to drive were identified as important predictors in eliciting citizens' preferences for measure design. However, for the willingness to support UVAR adoption, commuting mode, age, gender, income, perception of environmental effects of transport, and desire to shift to sustainable modes were the main determiners. The effect of socio-demographic variables across preferences for measure design and willingness to support the measures suggest the heterogeneity of residents’ preferences is an important consideration in UVAR measure planning. Hence, re-emphasising the importance of public participation and engagement at all phases of the UVAR measure planning and decision-making process. As recommended by [94], establishing an effective communication strategy that includes various engagement methods such as public consultations, surveys, and feedback mechanisms will be required. This can help gather diverse perspectives from the community and ensure their voices are heard in the decision-making process. Additionally, creating clear and accessible channels for information dissemination can increase transparency and build trust with the public. Finally, involving community leaders and stakeholders in the planning and decision-making process is crucial, as they can serve as advocates and bridge the gap between the represented urban communities and decision-makers.

## Supplementary Information

**Additional file 1: Table A.1**. Multiverse of Likelihood Ratio Tests Significance and goodness of fit parameters at different removal probability values for the stepwise multinomial logistic regression model. **Table A.2**. Multiverse of average importances for the survey complete responses and analytic sample.

## Data Availability

The datasets used or analysed during the current study are available from the corresponding author upon a reasonable request.

## Abbreviations

CI: Confidence interval
CO_2_: Carbon dioxide
COVID-19: Coronavirus disease of 2019
EU: European Union
EU-27: 27 Member states of the European Union
KMO: Kaiser–Meyer–Olkin
*p*: Probability of statistical measure
RLH: Root likelihood
S.E.: Standard errors of the individual regression coefficients for the estimated models
UVAR: Urban Vehicle Access Regulations
