# Supplementary material for: Preferences for policy measures to regulate urban vehicle access for climate change mitigation
Source: Environ Sci Eur. 2023 Jun 6;35(1):42. doi: 10.1186/s12302-023-00745-0 (PMC10241608; doi:10.1186/s12302-023-00745-0)
Supplement: Supplementary file 1 — Additional file 1: Table A.1. Multiverse of Likelihood Ratio Tests Significance and goodness of fit parameters at different removal probability values for the stepwise multinomial logistic regression model. Table A.2. Multiverse of average importances for the survey complete responses and analytic sample. [file 12302_2023_745_MOESM1_ESM.pdf]

Preferences for Policy Measures to Regulate Urban Vehicle Access for Climate Change Mitigation

Appendix – Multiverse Analysis

Table A.1. Multiverse of Likelihood Ratio Tests Significance and goodness of fit parameters at different removal probability values for the stepwise multinomial logistic regression model.

**Variables**	**Removal probability values**					
	**0.05**	**0.10**	**0.15**	**0.20**	**0.25**	**0.30**
Age	0.018	0.062	0.062	0.065	0.044	0.044
Gender	0.001	0.003	0.003	< 0.001	< 0.001	< 0.001
Education	-	-	-	0.164	0.139	0.139
Paid employment	-	-	-	-	-	-
Income	-	0.024	0.024	0.10	0.026	0.026
Origin	-	-	-	-	-	-
Destination	-	-	-	-	-	-
License	-	-	-	0.150	0.173	0.173
Commuting mode	-	0.097	0.097	0.095	0.182	0.182
Trip planning: Time	-	-	-	-	0.232	0.232
Trip planning: Cost	0.017	0.020	0.020	0.041	0.041	0.041
Trip planning: Environment	0.001	0.001	0.001	0.002	0.004	0.004
Problem perception: General transport	-	-	-	-	-	-
Problem perception: Environment	< 0.001	< 0.001	< 0.001	< 0.001	< 0.001	< 0.001
Problem perception: Driving	< 0.001	< 0.001	< 0.001	< 0.001	< 0.001	< 0.001
Behavioural intention: Reduce car usage	-	0.075	0.075	0.124	0.159	0.159
Behavioural intention: Change travel pattern	0.006	0.011	0.011	0.012	0.007	0.007
Behavioural intention: Modify car usage	0.008	0.015	0.015	0.030	0.032	0.032
Behavioural intention: Protest	< 0.001	< 0.001	< 0.001	< 0.001	< 0.001	< 0.001
**Summary statistics**						
Number of included variables	9	12	12	14	15	15
AIC	748.398	749.980	749.980	752.140	757.645	757.645
BIC	916.974	974.749	974.749	1000.990	1038.605	1038.605
McFadden R^2	0.239	0.269	0.269	0.280	0.292	0.292
Classification accuracy	63.3%	68.7%	68.7%	67.5%	67.5%	67.5%

Significances are shown for variables included in the model at the different threshold p-values

Table A.2. Multiverse of average importances for the survey complete responses and analytic sample

Attribute	Complete Responses (n = 471)			Analytic Sample (n = 409)		
	Average Importance	Lower CI	Upper CI	Average Importance	Lower CI	Upper CI
Policy coverage area	13.12	12.15	14.09	13.98	12.87	15.10
Policy effective period	17.38	16.48	18.28	16.12	15.18	17.06
Affected vehicles	14.81	13.84	15.78	14.29	13.22	15.37
Access Fee	20.90	19.72	22.30	22.14	20.59	23.69
Revenue allocation to transport development	20.24	18.85	21.63	20.75	19.53	21.97
Monitoring	13.54	12.66	14.42	12.72	11.80	13.64
NONE	-124.95	-161.56	-88.34	-162.45	-204.55	-120.34
**Fit Statistic (RLH)**	0.55			0.61		
